# SirT1 Regulates Energy Metabolism and Response to Caloric Restriction in Mice

**DOI:** 10.1371/journal.pone.0001759

**Published:** 2008-03-12

**Authors:** Gino Boily, Erin L. Seifert, Lisa Bevilacqua, Xiao Hong He, Guillaume Sabourin, Carmen Estey, Cynthia Moffat, Sean Crawford, Sarah Saliba, Karen Jardine, Jian Xuan, Meredith Evans, Mary-Ellen Harper, Michael W. McBurney

**Affiliations:** 1 Center for Cancer Therapeutics, Ottawa Health Research Institute, Department of Medicine, University of Ottawa, Ottawa, Ontario, Canada; 2 Department of Biochemistry, Microbiology and Immunology, University of Ottawa, Ottawa, Ontario, Canada; University of Arkansas, United States of America

## Abstract

The yeast sir2 gene and its orthologues in *Drosophila* and *C. elegans* have well-established roles in lifespan determination and response to caloric restriction. We have studied mice carrying two null alleles for SirT1, the mammalian orthologue of sir2, and found that these animals inefficiently utilize ingested food. These mice are hypermetabolic, contain inefficient liver mitochondria, and have elevated rates of lipid oxidation. When challenged with a 40% reduction in caloric intake, normal mice maintained their metabolic rate and increased their physical activity while the metabolic rate of SirT1-null mice dropped and their activity did not increase. Moreover, CR did not extend lifespan of SirT1-null mice. Thus, SirT1 is an important regulator of energy metabolism and, like its orthologues from simpler eukaryotes, the SirT1 protein appears to be required for a normal response to caloric restriction.

## Introduction

Silent information regulator 2 (sir2) is a yeast gene encoding a nicotinamide adenine dinucleotide (NAD^+^)-dependent histone deacetylase [1], [2]. Homologues of this protein are found throughout prokaryotes and eukaryotes [3]. In yeast, worms and flies, an extra copy of the sir2 gene or its orthologue increases lifespan by 18% to 50% [4]–[6] whereas a deletion of the gene, in yeast, reduces lifespan [4].

Mammals have 7 homologues of the Sir2 protein, Sirtuins 1-7 (SirT1-7) [7], [8]. SirT1 is the family member sharing the most homology to Sir2 [8] and is considered to be its orthologue. It is not known whether SirT1 is involved in aging although it is frequently assumed to share this role with its sir2 orthologues. SirT1 can deacetylate histones [1], [9] as well as a wide variety of nuclear and cytoplasmic proteins (reviewed in [10]).

We [11] and others [12] have created mice carrying targeted mutations in the SirT1 gene. On the 129/J inbred background, SirT1-null mice die shortly after birth; however, on an outbred genetic background, most SirT1-null mice survive to adulthood and some can reach 24 months of age. SirT1-null mice look grossly normal but are small, sterile, have craniofacial abnormalities, and develop an eyelid inflammatory condition [11].

Several lines of evidence suggest that SirT1 plays a role in energy metabolism. The dependence on NAD^+^ as a cofactor for catalysis is thought to link SirT1 activity to the energetic state of the cell [1], [2]. In mammals, glucose metabolism is regulated by insulin. SirT1 is known to promote insulin expression [13] and secretion [14], [15] in pancreatic β-cells and to modulate the expression [16] and secretion [17] of adiponectin, a hormone that enhances insulin sensitivity. The metabolism of glucose, fatty acids and cholesterol is modulated in various cell types by the effects of SirT1 on known regulators of metabolic enzymes such as PPARγ [18] and PGC-1α [19]–[21].

Calorie restriction (CR) has been known for decades to extend lifespan of virtually all organisms from yeast to mammals. Sir2 is required for CR to mediate lifespan extension in yeast and flies [6], [22]. We set out to determine if SirT1 is required for CR to mediate its physiological response in mammals by studying the energy metabolism of SirT1 null-mice under ad libitum (AL) or CR diets. We report below that SirT1-null mice inefficiently utilize food, are hypermetabolic and that their liver mitochondria are functionally altered. Importantly, we present data suggesting that SirT1 is required for *in vivo* response to CR.

## Results

### Energy balance in SirT1- null mice

**Figure 1:**
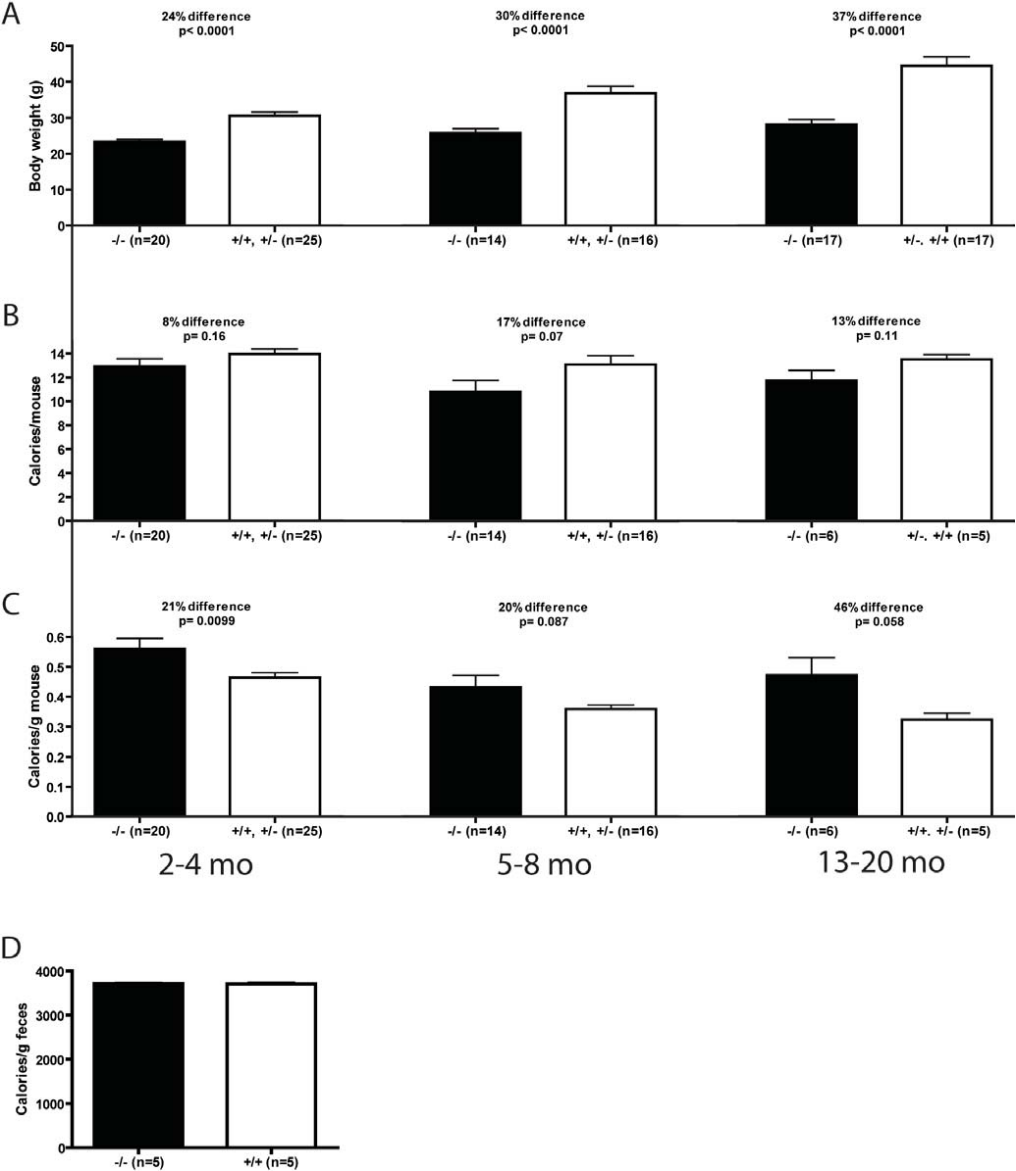
SirT1-null mice are hyperphagic. A. Body weights of sibling animals of normal (SirT1^+/+^ and SirT1^+/−^) and SirT1(^−/−^)-null genotypes at 2–4, 5–8 and 13–20 months of age. B. Daily food intake of mice as described in A. C. Daily food intake normalized to body weight. D. Caloric content of feces from 2–4 months old normal and SirT1-null mice. Means and standard errors are represented along with the number of animals used for each determination. Unpaired T-tests were performed to assess statistical significance.

We have previously created mice that do not synthesize the SirT1 protein [11]. Early in their life (2–4 months old), SirT1-null mice are about 25% smaller than their normal littermates (Figure 1A). As they get older, this difference increases to 30% at 5–8 months and almost to 40% at 13–20 months (Figure 1A). Despite this difference in size, SirT1-null mice eat similar or only slightly less food than controls (Figure 1B). When daily food intake is normalized to body weight (BWT), it is evident that the SirT1-null mice are hyperphagic (Figure 1C). This difference in food intake is not caused by inefficient food absorption by the digestive tract as bomb calorimetry of fecal material from SirT1-null and normal mice were equally depleted of calories (Figure 1D).

**Figure 2:**
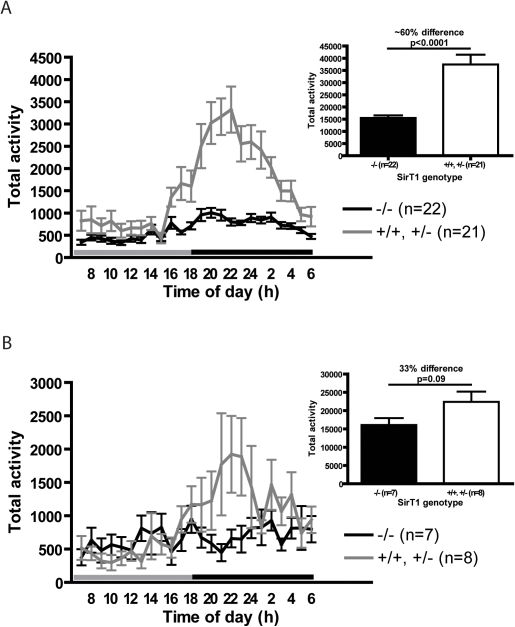
SirT1-null mice are less active than controls. Activity during 60-minute intervals of individually caged SirT1-null and normal littermates 3–6 months (A) and 9–12 months (B) of age. Insets show histograms of total activity in a 24 hour period. Grey and black lines above the X-axis indicate light and dark periods, respectively. Means and standard errors are represented. Unpaired T-tests were performed to assess statistical significance.

The low body mass and hyperphagia of the SirT1-null mice might be a consequence of enhanced activity but we found that young SirT1-null mice are much less active than their normal littermates, particularly during the dark period (Figure 2A). As these mice got older, the difference between the genotypes decreased primarily because the activity of normal animals declined (Figure 2B). Thus the small and hyperphagic SirT1-null mice are also lethargic compared to their normal littermates suggesting that there is inefficient usage of ingested calories in these animals.

### Hormonal profile of SirT1-null mice

**Figure 3:**
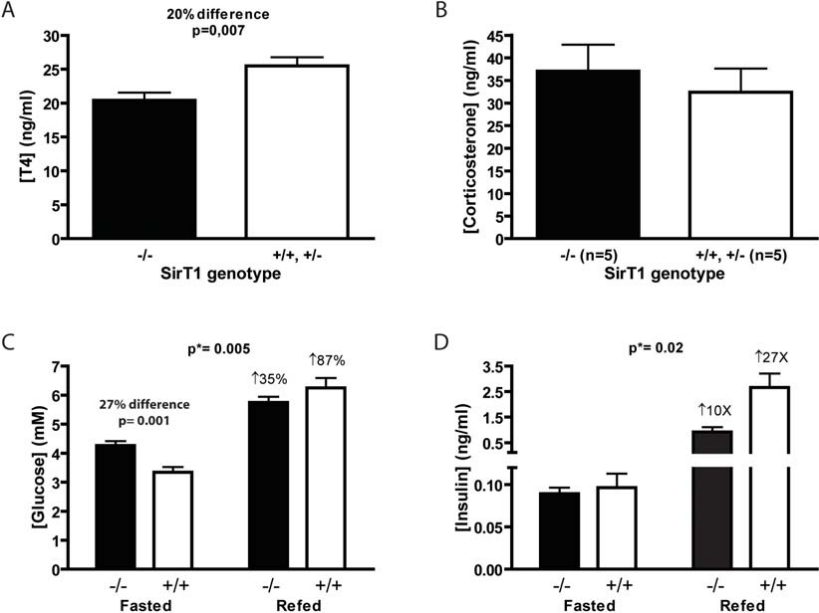
Serum thyroxin (T4), corticosterone, glucose and insulin levels. A. T4 levels were measured from serum samples of SirT1-null and normal mice using an EIA kit. B. Corticosterone levels were measured from plasma samples of SirT1-null and normal mice at 4 hours intervals during the diurnal cycle using an EIA kit. The peak values are plotted. Unpaired T-tests were performed to assess statistical significance. Blood glucose (C) and insulin (D) levels of 2–3 months old mutant and wild type mice were measured after 24 hour fasting and again 3 hours after refeeding using a standard glucometer (C) and an ELISA kit (D). Means and standard errors are represented. Two-way ANOVAs was performed to assess statistical significance of interaction between genotype and dietary condition (p*-values on top of panel), and unpaired T-test to assess difference between genotypes within dietary condition groups (p-value over bars).

Systemic energy metabolism is under the control of hormones. Thyroxine (T4) levels were slightly lower in the SirT1-null serum (Figure 3A) whereas peak corticosterone levels were normal (Figure 3B). Fasted glucose levels of SirT1-null mice were higher than those of normal littermates (Figure 3C) but insulin concentrations were not different (Figure 3D). Three hours after re-feeding, the insulin levels of normal animals rose by nearly 30 times whereas the rise in SirT1-null mice was increased only 10 fold (Figure 3D). The postprandial blood glucose concentration was similar or lower in SirT1-null compared to normal mice (Figure 3C), suggesting that SirT1-null mice might be insulin sensitive. A similar result was observed previously [15]. However, insulin tolerance tests did not reveal a difference between SirT1-null and normal animals (data not shown). Blood lactate concentrations were also not different between normal and SirT1-null mice suggesting that there was no loss of food energy through excessive glycolysis (data not shown).

### Indirect calorimetry

**Figure 4:**
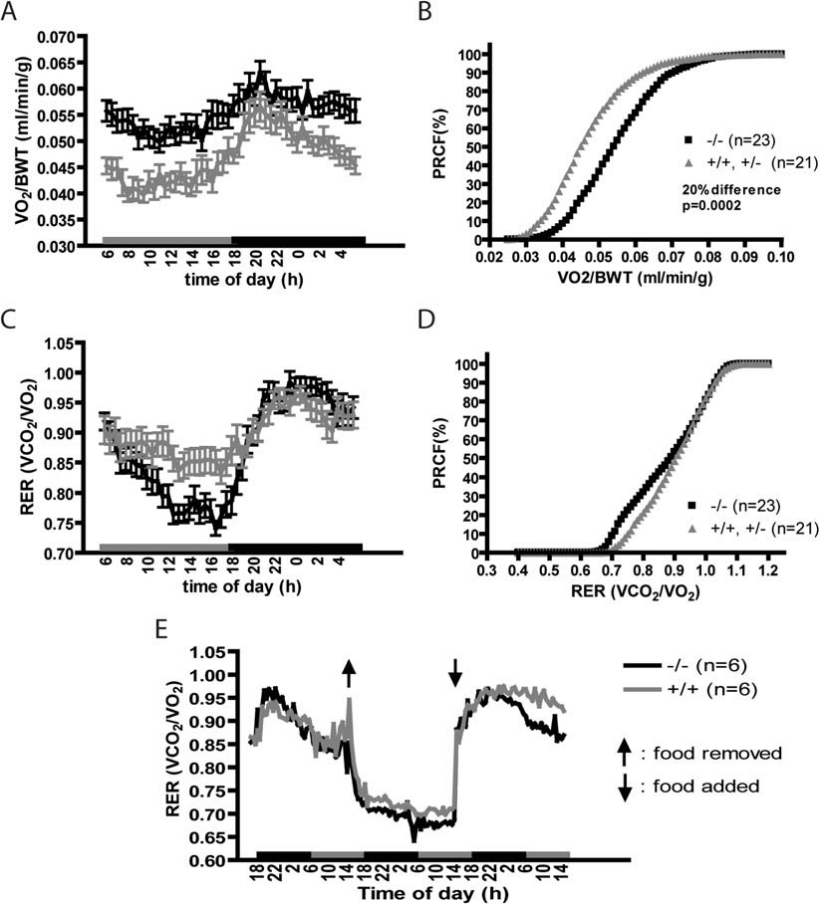
SirT1-null mice are hypermetabolic. Whole animal indirect calorimetry (IC) was used to assess oxygen consumption normalized to body weight (VO_2_/BWT) plotted (panel A) at 30 minute intervals during a 24 hour period or (panel B) as the percent relative cumulative frequency (PRCF) of VO_2_/BWT. The respiratory exchange ratio (RER = VO_2_/VCO_2_) was calculated from VO_2_ and VCO_2_ data and plotted at 30 minute intervals during a 24 hour period (panel C) or as PRCF (panel D). In a fasting-refeeding experiment (panel E), food was either removed or added at the indicated times (arrows) and the interval RER plotted. Grey and black lines above the X-axis (panels A, C, and E) indicate light and dark periods, respectively. An RER of 1.0 is expected for glucose oxidation and an RER of 0.7 occurs during lipid oxidation. Means and standard errors are represented. An unpaired T-test using medians was performed to assess statistical significance (B).

To estimate metabolic rate and substrate utilization, we measured oxygen consumption and carbon dioxide production from whole animals. When normalized to body weight, oxygen consumption of SirT1-null mice was higher than that of controls, indicating that they are hypermetabolic (Figure 4A, B), a conclusion consistent with their normal food ingestion and lower body mass. The largest difference in oxygen consumption occurred during the light period when both SirT1-null and normal mice are relatively inactive. Respiratory exchange ratio (RER = VCO_2_/VO_2_) indicated that SirT1-null animals rely more on lipid substrates (RER values close to 0.7) during the 6–8 hours before the onset of the dark period, the time when they will have their main meal (Figure 4C). This increased fatty acid oxidation in SirT1-null mice is also apparent when data are plotted as percent relative cumulative frequencies (PRCF), as indicated by the shoulder of the mutants' curve around RER values of 0.7–0.8 (Figure 4D). We measured free fatty acids in the serum of mice fasted overnight and found that the concentrations tended to be lower in SirT1-null mice than in normal mice; however, this difference was not statistically significant (data not shown).

It has been proposed that SirT1 is required for the induction and maintenance of fatty acid oxidation in response to low glucose concentration [19]. However, SirT1-null mice appear to readily switch between glucose to lipid utilization for oxidative phosphorylation (Figure 4C). To further test their ability to switch substrates from glucose to lipids, we performed fasting/refeeding experiments. When mice are fasted, RER drops to 0.7 as lipids are used as oxidation substrates and following refeeding, RER climbs to 1.0 as glucose becomes the predominant energy source. SirT1-null mice switch readily between lipid and glucose substrates as evidenced by the efficient changes in RER (Figure 4E). Interestingly, the day after refeeding, RER in SirT1-null animals started to decline much faster than that in normal animals suggesting limited glucose utilization even when food is freely available.

**Figure 5:**
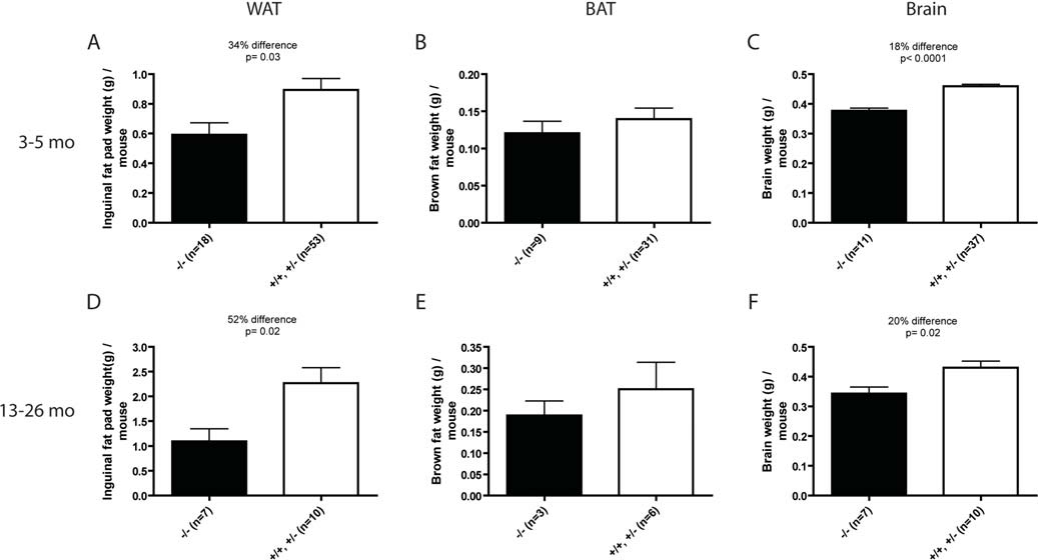
Organ weights of SirT1-null mice. Organs from 3–5 month (A–C) and 13–26 month old mice (D–F) were weighed and plotted. Inguinal fat pad (A, D), interscapular BAT (B, E) and brain (C, F) are plotted as means and standard errors. Unpaired T-tests were performed to assess statistical significance.

The observation that SirT1-null mice have higher rates of lipid oxidation than normal is consistent with previous observations that in older SirT1-null mice the lipid content of cells in white adipose tissue (WAT) are smaller than those of normal tissues [23]. We compared the total weight of some lipid storage tissues in young and old mice. In young animals, the inguinal fat pads from SirT1-null mice were smaller than those of their littermates (Figure 5A) and this tissue increased in mass with age much more slowly than normal in SirT1-null mice (Figure 5D). The interscapular BAT depot was similar in size in young and in older mice (Figure 5B, E) perhaps reflecting its role in energy dissipation. The brain weight of SirT1-null mice is about 20% smaller than normal mice and in both young and older animals (Figure 5 C, E). These results coupled with observations noted above [23] are consistent with the notion that SirT1-null mice burn stored lipids at rates in excess of those in normal animals.

### Liver mitochondria from SirT1-null mice are less efficient than normal

**Figure 6:**
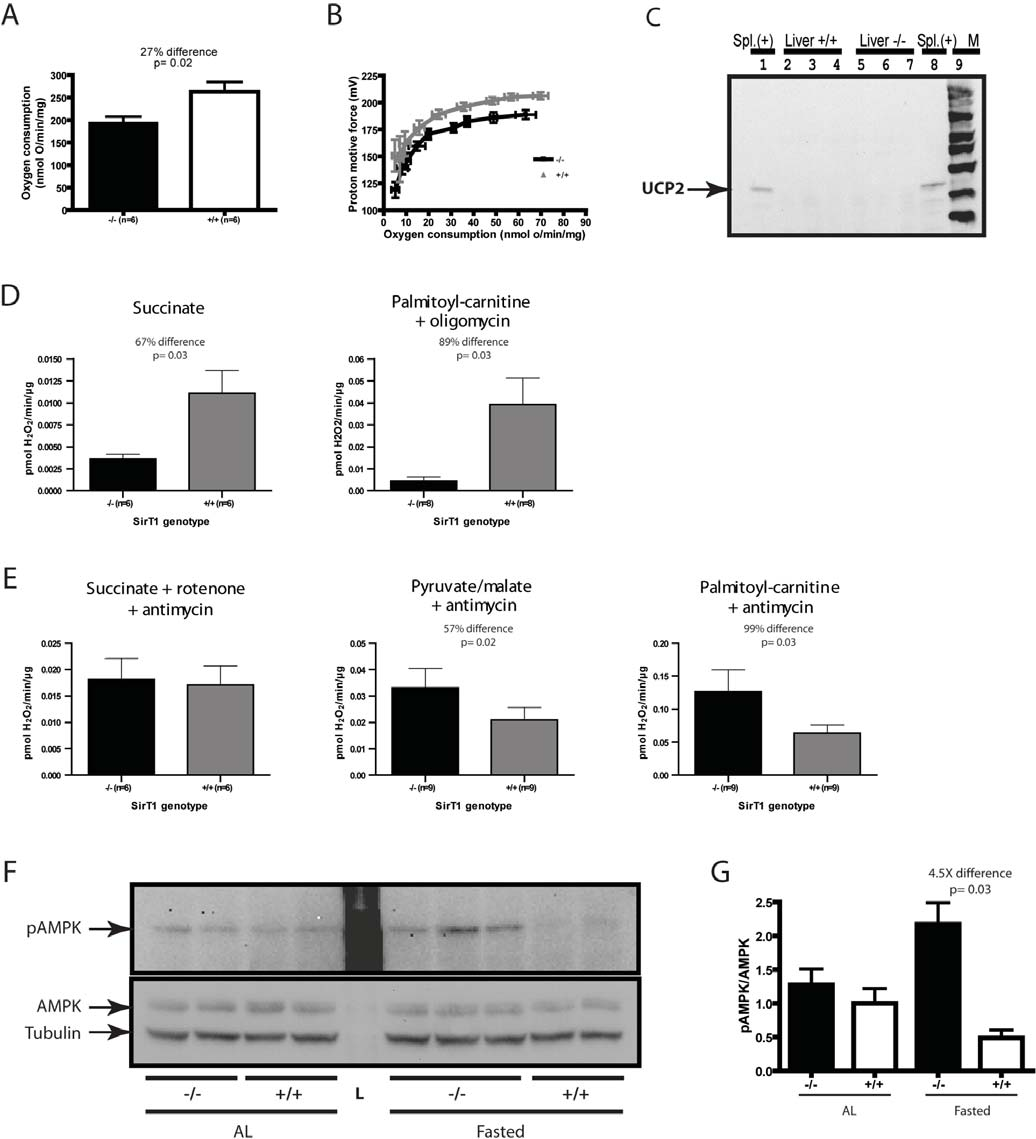
Respiration and ROS production of isolated mitochondria. A. State 3 oxygen consumption rate of liver mitochondria from SirT1-null and normal mice was determined using succinate, in the presence of rotenone. Proton motive force (PMF) was determined in liver mitochondria (B) using succinate, in the presence of rotenone and a saturating amount of oligomycin. The farthest point on the right represents the maximal state 4 oxygen consumption rates. The kinetic response of PMF was determined by inhibiting respiration targeting complex II by incremental additions of malonate (up to 5 mM). C. Western blot of UCP2 from liver mitochondria. Thirty µg of mitochondria-enriched proteins from normal spleen (Spl., positive control) or liver from 3 different SirT1-null and normal mice were loaded separately in each lane, electrophoresed, blotted and probed with an antibody to UCP2. Note that UCP2 protein expression is not de-repressed in mutants. D–E. ROS production from liver mitochondria was measured using the p-hydroxyphenylacetate (PHPA) assay using the substrates and respiration inhibitors indicated at the top of each column. F. Western blot of phospho-AMPKα (pAMPK), AMPα (AMPK), and α−tubulin. Hundred-fifty µg of liver proteins from 2–3 different SirT1-null and normal mice under the indicated dietary condition was loaded separately in each lane, electrophoresed, blotted and probed with an antibody to pAMPK. Membrane was stripped and reprobed sequentially with antibodies to AMPK and α−tubulin. G. Densitometry of western blot signal in F. Bands in F were quantified by densitometry using the ImageJ software. Means and standard errors are represented. Unpaired (A, G) or paired (D–E) T-tests were performed to assess statistical significance (see Materials and Methods).

SirT1-null mice are hypermetabolic but lethargic, suggesting that their energy generation system might be defective. We investigated the state 3 (maximal phosphorylating) respiration rate as well as proton conductance in isolated mitochondria from liver and skeletal muscle. We detected no significant differences in the mitochondria from skeletal muscle (data not shown). However, the rate of respiration under state 3 conditions was lower in SirT1-null liver mitochondria suggesting that these mitochondria would produce less ATP at full capacity than those from normal mice (Figure 6A). Measurement of proton motive force under state 4 (non-phosphorylating) respiration conditions (Figure 6B) revealed that it was lower in SirT1-null liver mitochondria. This suggests that the inner membranes of the SirT1-null mitochondria are relatively permeable to protons and that less proton motive force would be generated to produce ATP.

Uncoupling proteins such as UCP2 can diminish proton motive force and SirT1 is known to inhibit the expression of ucp2 in pancreatic beta-cells [15]. The level of ucp2 mRNA was measured in liver from SirT1-null and normal mice but no difference in abundance was detected (data not shown). Western blots (Figure 6C) indicated that no UCP2 protein was detectable in mitochondria from either normal or SirT1-null liver.

In addition to ATP synthesis, mitochondria are the major site for the generation of reactive oxygen species (ROS). We measured the capacity of liver mitochondria to produce H_2_O_2_ under several conditions. In the presence of succinate or palmitoylcarnitine, liver mitochondria from SirT1-null animals produced less H_2_O_2_ than normal (Figure 6D), consistent with the observation that these mitochondria have a higher proton leak (see Figure 6B and [24], [25]).

We also took advantage of this H_2_O_2_ production assay as an indirect method to probe the capacity of pathways upstream of the electron transport chain (ETC) to provide reducing equivalents (RE) to the ETC. Mitochondria were subjected to three different substrates (succinate, pyruvate+malate or palmitoylcarnitine) in the presence of antimycin so that H_2_O_2_ would be produced predominantly from complex III in all conditions. Succinate feeds into complex II directly, so the H_2_O_2_ produced using this substrate is not affected by the capacity of any pathways upstream of the ETC to provide RE. Mitochondria from both normal and SirT1-null liver produced similar levels of H_2_O_2_ under this condition (Figure 6E). On the other hand, pyruvate+malate and palmitoylcarnitine first need to be oxidized through beta-oxidation and/or the TCA cycle, so the H_2_O_2_ produced using these substrates is affected by the capacity of these upstream pathways to provide reducing equivalents to the ETC. SirT1-null mitochondria produced higher levels of H_2_O_2_ when using pyruvate+malate or palmitoylcarnitine (Figure 6E), suggesting that SirT1-null mitochondria have higher than normal beta-oxidation and TCA cycle capacities. These results are consistent with an increased lipid utilization in SirT1-null mice.

AMP-activated protein kinase (AMPK) is a sensor for the availability of energy in cells. Our observations with SirT1-null liver mitochondria suggest that they are less efficient than normal in producing ATP. We measured the levels of the active form of AMPK, phopho(Thr172)-AMPKα (pAMPK), in liver from mice fed AL or fasted for 24 hours. Similar levels of activated pAMPK were observed in liver from AL-fed mice, regardless of the genotype (Figure 6F–G). Normal mice had reduced pAMPK after 24 hour fasting while SirT1-null liver had a 2-fold increase in pAMPK signal (Figure 6F–G). These data suggest that liver from SirT1-null mice maintains normal ATP levels under AL conditions but fails to maintain ATP levels during fasting.

### SirT1 is required for CR response in mammals

Sir2 is required for lifespan extension in *Saccharomyces cerevisiae* [22] and *Drosophila melanogaster* [6] when exposed to caloric restriction (CR). To determine whether SirT1 is also required for CR response in mammals, we subjected 5 to 7 month old SirT1-null mice to a diet with 40% reduced calorie content for up to 44 weeks and compared their response with that of their normal littermates.

**Figure 7:**
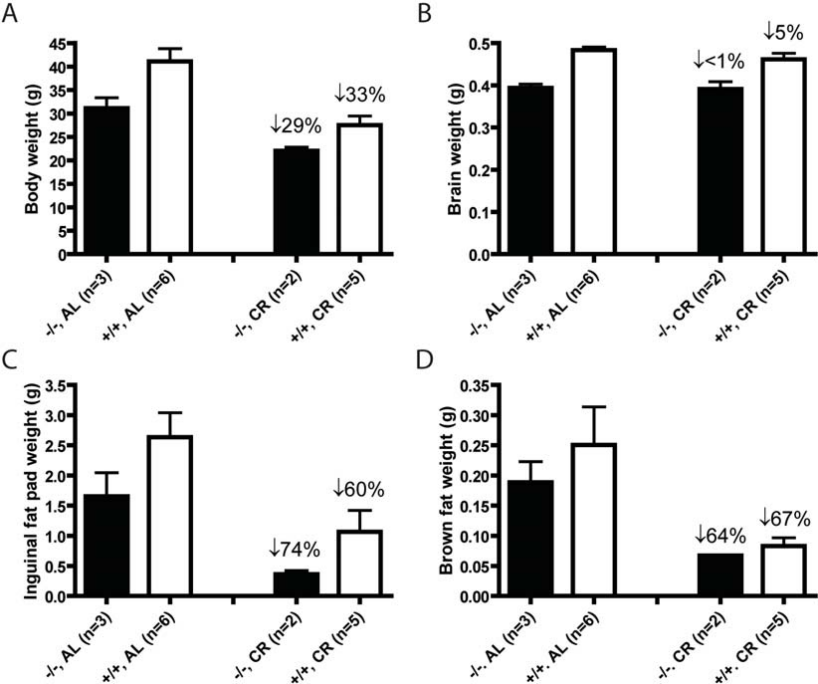
Whole body and organ weights following CR. The weights of whole body (A), brain (B), inguinal fat pad (C), and interscapular BAT (D) were obtained from 13–15 months old mice, AL-fed or CR for 25 to 28 weeks. Percentages above bars represent the percent reduction in weight compared to AL-fed mice.

CR resulted in a reduced body weight that was similar in both normal and SirT1-null animals (Figure 7A). Brain did not show any decrease in weight with CR (Figure 7B) but the inguinal fat pad weight was reduced more dramatically in the SirT1-null mice than in normal animals (Figure 7C). Brown fat was reduced to similar degrees in SirT1-null and normal mice (Figure 7D). These data suggest that SirT1-null mice rely more on lipid mobilized from WAT than normal mice, an observation consistent with the higher lipid utilization of SirT1-null animals under AL conditions. SirT1 protein in heart, liver, BAT and WAT of CR treated normal mice was only marginally, but not significantly, increased compared to AL-fed mice (data not shown), a result different from that previously reported [26].

**Figure 8:**
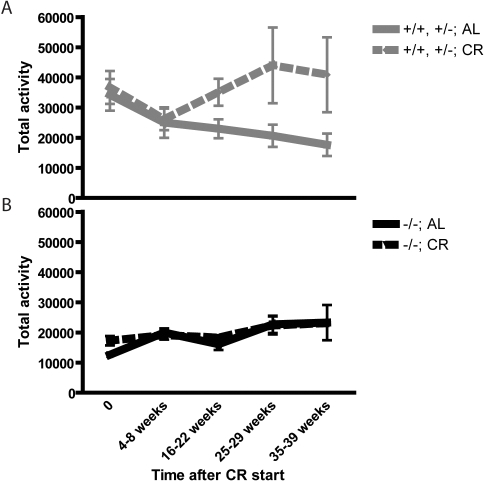
Activity of mice during CR. Total activity of normal (A) and SirT1-null (B) mice was monitored using a Micromax system for 24-hour periods at the indicated time after the start of CR. Animals were 5–7 months old at the beginning of CR. Means and standard errors are represented. The activity of CR normal mice increased with time whereas that of AL controls decreased. CR did not alter the activity of SirT1-null mice.

The daily cumulative physical activity of normal animals increased with CR, as previously reported [27], while that of AL fed normal mice declined with age (Figure 8A). In contrast, the physical activity of SirT1-null mice did not increase with CR but remained similar to that of AL fed animals (Figure 8B).

**Figure 9:**
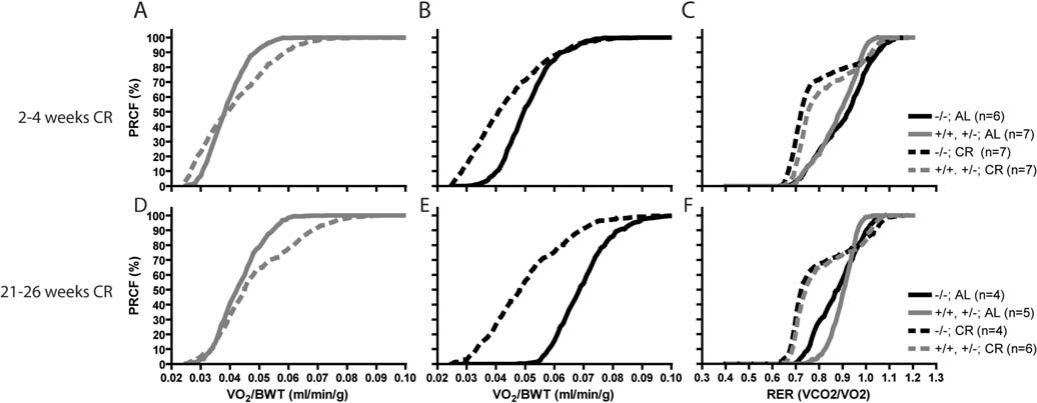
Whole animal oxygen consumption during CR. Oxygen consumption normalized to whole body weight (VO_2_/BWT) for normal (A, D) and SirT1-null (B, E) mice was measured after 2–4 weeks (A–C) and 21–26 weeks (D–F) of CR or AL diet as indicated. RER from the same animals at 2–4 weeks (C) or 21–26 weeks (F) of CR were also obtained. Data were plotted as PRCF as in Figure 4. The normal mice adapted to CR by maintaining their levels of oxygen consumption but the oxygen consumption of SirT1-null animals was higher in AL than in CR. This difference was particularly evident in older SirT1-null mice (panel E) when AL animals' oxygen consumption was very high.

Despite a 40% reduction in caloric intake, body weight-normalized oxygen consumption of normal mice subjected to CR was the same or slightly higher than AL fed animals (Figure 9A, D). The situation with SirT1-null animals was different. Oxygen consumption of SirT1-null mice was significantly lower in CR animals compared to AL fed mice (Figure 9B, E). We interpret this to indicate that the SirT1-null mice are unable to adapt to the reduced calorie intake and consequently their metabolic rate is reduced. RER values of SirT1-null and control mice under CR were similarly low with values close to 0.7 (∼70% of all values), indicating a preferential oxidation of lipids (Figure 9C, F).

**Figure 10:**
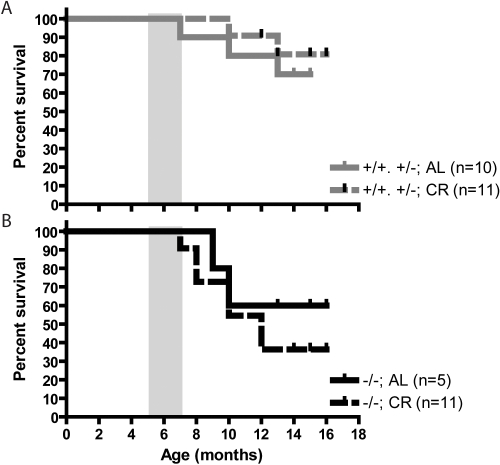
CR had no beneficial effect on survival of SirT1-null mice. Survival curve for normal (A) and SirT1-null (B) mice that were fed the CR of AL diets. Mutant mice that were euthanized because of eyelid inflammation were excluded from this graph. Grey rectangles represent the period when CR started (5 to 7 months old).

The SirT1-null animals are less fit than normal and many fail to survive the first year following birth. As CR is a well-established means of prolonging the lifespan of mice, we looked at the survival of SirT1-null and normal mice during our CR experiments. These experiments involved relatively few animals but the data seem to indicate that the decreased viability of SirT1-null mice was exacerbated during CR (Figure 10B). Normal animals were too young to suffer significant loss of viability during the course of this experiment so we have no evidence for an effect of CR on their lifespan (Figure 10A).

## Discussion

Since its catalytic activity is dependent on NAD^+^, SirT1 deacetylase activity has been postulated to be controlled by the metabolic state of the cell [1], [2]. In fact, our observations suggest that SirT1 is an important regulator of metabolic activity because SirT1-null mice utilize ingested calories inefficiently and because SirT1-null mice do not adapt normally to CR or to fasting.

SirT1-null mice are smaller and lethargic compared to their normal littermates but, per gram of body weight, they consume more food and oxygen. Thus, the SirT1-null animal is metabolically inefficient compared to normal. Liver mitochondria appear to have a lower capacity to produce ATP because of lower state 3 respiration capacity and higher proton leak through the inner mitochondrial membrane. Perhaps to compensate for their reduced oxidative phosphorylation capacity, mitochondria from SirT1-null liver have increased capacity for TCA cycle and beta-oxidation. This compensation seems to be successful in maintaining the level of ATP but overnight fasting resulted in much higher levels of phospho-AMPK suggesting that ATP levels are not sustained in SirT1-null mice in the face of food deprivation.

SirT1 is known to modulate the activities of several regulators of metabolism but an examination of the characteristics of the SirT1-null mouse and the expectations based on published studies yields a number of unexpected contradictions. As a negative regulator of PPARγ, SirT1 decreases transcription of genes involved in fat storage [18] so the SirT1-null mice would be expected to have increased WAT depots; in fact WAT is reduced in these mice. SirT1 is a positive regulator of PGC1α [21], [28], so one might expect that tissues from SirT1-null mice would have fewer mitochondria. This does not seem to be the case as determined by measurements of the mitochondrial DNA copy number. Recent reports have indicated that SirT1 regulates insulin signaling either directly by deacetylation of IRS2 [29] or indirectly by derepressing PTP1B [30]. Both mechanisms would predict that SirT1-null mice should be insulin resistant. We found that the SirT1-null animals showed normal insulin-dependent glucose regulation. SirT1 is reported to be required for the switch from glucose to lipid oxidation [19] but SirT1-null mice had no difficulty in making this switch; in fact, these animals had a higher level of lipid oxidation under AL feeding conditions. Because SIRT1 is expressed in all tissues it is difficult to extrapolate information obtained from isolated cells and organs to accurately make predictions regarding the intact animal.

The means by which CR extends lifespan is not yet clear. In yeast and *D. melanogaster*, Sir2 has been shown to be required for CR to increase lifespan [6], [22]. Here we show that SirT1 is also required in mammals for some responses to CR. First, we confirmed that the CR-mediated increase in physical activity is not observed in SirT1-null mice [27]. Second, we show that, whereas normal mice maintain their metabolic rate when subject to CR, that of the mutant mice drops dramatically. Finally, SirT1 appears to be required for CR to increase lifespan. The beneficial effects of CR on lifespan of normal mice is typically observed after 20 months of age (e.g. [31]). Because our CR experiment was terminated before animals reached this age, we saw no difference in survival between our CR and AL groups of normal mice. However, we had already lost two-thirds of our SirT1-null mice by the end of this experiment and CR had no obvious beneficial effect on lifespan of these mice. In fact, CR seemed to augment the early demise of SirT1-null mice. We interpret our data to indicate that SirT1-null mice are not capable of adapting to CR conditions.

Lifespan is thought to be determined by the accumulation of cellular damage arising from ROS although recent evidence from C. elegans suggests that ROS might in fact be responsible for extending lifespan (Schulz et al., 2007). It is perhaps relevant that SirT1-null mice have liver mitochondria that produce less ROS than normal. In regards to mitochondrial uncoupling, this observation is consistent with increased proton leak in these mitochondria; however, the increased leak is not due to a derepression of ucp2, as one could infer from studies in pancreatic beta-cells [15]. Nevertheless, there are a number of other regulators of mitochondrial activity yet to be investigated [32], [33].

In conclusion, our study indicates that the absence of SIRT1 results in a metabolically inefficient animal that fails to adapt to CR conditions. Given that there are over 30 SirT1 substrates to date [10], it is remarkable that SirT1-null mice are viable and can sometimes reach 2 years of age. At this point, it is not yet clear which of the many SIRT1 substrates is/are responsible for the phenotype or which tissue(s) share the metabolic defect. A better understanding of the role of SirT1 in energy metabolism may help in designing strategies to provide the health benefits of CR without curtailing dietary energy intake.

## Materials and Methods

### Animals

All animal experiments were performed according to the Guidelines for the Care and Use of Animals established by the Canadian Council on Animal Care. The mutant mice used in this study carried the *sirt1*-null allele previously described [11] maintained on a mixed genetic background derived from intercrosses between the CD1 out bred strain and 129/J. SirT1-null animals were created by crossing heterozygotes and were identified at weaning by a characteristic eyelid defect. The genotypes of animals were determined by a PCR-based test carried out on DNA isolated from tail tip biopsy. The primers TTCACATTGCATGTGTGTGG and TAGCCTGCGTAGTGTTGGTG amplify a 423 bp fragment from the normal *sirt1* allele while a 526 bp fragment from the null allele is amplified from the first primer and ATTTGGTAGGGACCCAAAGG, a sequence derived from the *pgk-1* gene inserted to create the null allele by homologous recombination. *sirt1*-null mice were normally housed in cages with littermates of the same sex.

### Feces and bomb calorimetry

Two to four months old mice were caged individually in metabolic chambers and feces were collected after 48 to 72 hours. Feces were dehydrated in a speedvac at 50°C overnight and grounded to powder. Gross energy (GE) of feces was determined using an automatic bomb calorimeter (Parr, 1271, Parr Instruments, Moline, IL, USA).

### Activity monitoring

Mice were caged individually and activity was recorded for 24-hour periods in a MicroMax activity monitoring system with 16 infrared beams per cage (AccuScan Instruments, Columbus, OH, USA). Total activity data were used for analyses. Lighting was on a normal 12 h light/dark cycle.

### Indirect calorimetry

Mice were caged individually and oxygen consumption (VO_2_) and carbon dioxide production (VCO_2_) were measured using a four-chamber Oxymax system with automatic temperature and light controls (Columbus Instruments, Columbus, OH). Temperature was maintained at 24°C, and lighting was on a normal 12 h light/dark cycle. System settings included a flow rate of 0.5 L/min, a sample line-purge time of 2 min, and a measurement period of 60 s every 12 minutes. The respiratory exchange ratio (RER) was calculated as the ratio of VCO_2_ produced/VO_2_ consumed.

### Isolation of mitochondria

Four to 5 month old mice were euthanized by decapitation for isolation of liver and skeletal muscle mitochondria. All media were ice-cold, and the procedures done on ice or at 4°C. Isolation of skeletal muscle mitochondria was performed using a modified method of Chappell and Perry [34]. Briefly, the skeletal muscles (pectoral, forelimb and hind limb muscles) were quickly dissected and placed in basic medium for muscle (BMM: 140 mM KCl, 20 mM HEPES, 5 mM MgCl_2_, 1 mM EGTA; pH 7.0). Muscle was cleaned of visible connective tissue and fat, minced by razor blade and placed in 15 volumes of homogenizing medium (HMM: BMM supplemented with 1 mM ATP and 1% BSA (w/v)). One unit of protease (Subtilisin A, Sigma, Oakville, ON, Canada) per g tissue wet weight was added to the muscle mixture. Tissue was homogenized using a glass/Teflon Potter-Elvehjem tissue grinder and fractionated by centrifugation at 800 g (10 min). The supernatant was collected and respun at 12,000 g (10 min). The resulting pellet was resuspended in BMM and incubated on ice for 3 min to allow myofibrillar repolymerization. Samples were spun at 800 g (10 min), the supernatant collected then spun at 12,000 g (10 min). The final pellet was resuspended in 220 µl of BM. Liver mitochondria were isolated as described [35]. Briefly, the liver was rapidly dissected and immersed in ice-cold basic medium for liver (BML: 250 mM sucrose; 10 mM Tris-HCl; 1 mM EGTA; pH 7.0), minced, then homogenized in HML (BML plus 0.5% de-fatted BSA) using a glass/Teflon Potter-Elvehjem tissue grinder. Liver homogenate was centrifuged at 800 g (4 min), followed by a centrifugation of the supernatant at 12,000 g for 7 min to pellet mitochondria. The pellet was washed twice in BML. The final pellet was resuspended in 250–400 ul of BML. Protein concentration was determined using a modified Lowry method with BSA as the standard.

### Mitochondrial respiration

Oxygen consumption was measured in isolated mitochondria (0.5 mg/ml) at 37°C using a Clark-type oxygen electrode (Hansatech, Norfolk, UK) and incubated in standard incubation medium (IM: 120 mM KCl, 1 mM EGTA, 5 mM KH_2_PO_4_, 5 mM MgCl_2_ and 5 mM HEPES; pH 7.4) containing 0.3% defatted BSA and assumed to contain 406 nmol O/ml at 37°C [36]. State 3 (maximum phosphorylating) respiration was determined using 10 mM succinate as substrate, and 250 µM ADP and 5 µM rotenone. State 4 (non-phosphorylating, or maximal leak-dependent respiration) was determined following addition of oligomycin (12 µg/mg protein). All measurements were done in duplicate.

### Mitochondrial protonmotive force

A methyl-triphenyl-phosphonium (TPMP^+^)-sensitive electrode was used to assess mitochondrial protonmotive force (Δp) [37]–[39] in non-phosphorylating skeletal muscle and liver mitochondria (see above). The TPMP^+^ electrode was calibrated by sequential 1 µM additions of TPMP^+^. Nigericin (80 µn/ml) was added to convert the pH component of Δp into mV units. The kinetics of proton conductance was assessed by incremental addition of malonate (up to 5 mM). After each run, 0.2 µM FCCP was added to release TPMP^+^ for baseline correction. TPMP^+^ measurements were done in triplicate and simultaneous with oxygen consumption determinations.

### Mitochondrial H_2_O_2_ production capacity

Mitochondrial H_2_O_2_ production rate was determined in freshly isolated mitochondria from liver using the *p*-hydroxyphenylacetate (PHPA) fluorometric assay [35], [40]. Mitochondria (0.6 mg/ml) were incubated in IM supplemented with 0.3% defatted BSA. H_2_O_2_ production was determined using pyruvate/malate (10 mM/5 mM; complex I-linked substrate), succinate (10 mM; complex II-linked substrate), or palmitoylcarnitine (20 µM; lipid-derived substrate that feeds into both complex I and II), under various conditions: 1) succinate, to assess H_2_O_2_ production generated by reverse electron flow through complex I, which is highly sensitive to uncoupling of oxygen consumption from oxidative phosphorylation, *e.g.* through the activation of uncoupling proteins [24]; 2) palmitoylcarnitine in the presence of oligomycin, to assess H_2_O_2_ production in resting mitochondria; 3) succinate in the presence of rotenone (5 µM) and antimycin (10 µM) to assess H_2_O_2_ production with full reduction of complexes I and III; 4) pyruvate/malate in the presence of antimycin (10 µM) to assess H_2_O_2_ production with full reduction of complex III and 5) palmitoylcarnitine in the presence of antimycin (10 µM) to assess H_2_O_2_ production with full reduction of complex III. All measurements were performed in the presence of superoxide dismutase (100 U/ml) to convert extra-mitochondrially-released superoxide to H_2_O_2_. H_2_O_2_ production was monitored for up to 25 min using a temperature-controlled fluorimeter (BioTek, FLx800) at 37°C. Fluorescence readings were converted to H_2_O_2_ production rates by use of a standard curve. Data were normalized to amount of proteins. To control for day-to-day variability, paired T-tests were used to determine statistical significance (1 SirT1-null and 1 WT were used simultaneously on the same day).

### Immunoblots

For the evaluation of UCP2 levels, liver mitochondria from SirT1 mutant and WT mice, as well as spleen mitochondria from WT mice, were isolated (see “Isolation of mitochondria” section above) and 30 µg of protein was loaded on a NuPAGE Bis-Tris 4–12% gradient precast polyacylamide gel (Invitrogen, Burlington, ON, Canada), electrophoresed, blotted on a nitrocellulose membrane and probed with an antibody to UCP2 (C20) (cat. #: sc-6525, Santa Cruz Biotechnology, Santa Cruz, CA, USA). Spleen was used as a positive control for UCP2 expression. Loading was verified by Ponceau Red staining.

For evaluation of phospho-AMPKα and AMPKα levels in liver tissue, SirT1 mutant and WT mice were euthanized by cervical dislocation, and pieces of liver were harvested and flash frozen within 1 min. Tissues were homogenized in ice-cold RIPA-phosphatase inhibitor buffer (Tris 20 mM pH 8.0, NP-40 1%, sodium deoxycholate 0.25%, NaCl 150 mM, EDTA 1 mM, Complete protease inhibitor cocktail 1× (Roche, Laval, QC, Canada), Na_3_VO_4_ 1 mM and NaF 1 mM) using a polytron homogenizer. Samples were centrifuged in a microfuge at 4°C, at full speed for 10 min and supernatants were collected and frozen at −80°C. A hundred fifty µg of proteins of each sample were loaded on a NuPAGE Bis-Tris 4–12% gradient precast polyacylamide gel (Invitrogen, Burlington, ON, Canada), electrophoresed, blotted on a nitrocellulose membrane and probed overnight at 4°C with an antibody to phospho-AMPKα (Thr172) (cat. #: 2531, Cell Signalling Technology, Danvers, MA, USA). Membrane was stripped in stripping buffer (SDS 2%, Tris 62.5mM pH 6.8, β-mercaptoethanol 100 mM) at 55°C for 30 min, washed, blocked and reprobed overnight at 4°C with an antibody to AMPKα (23A3) (cat. #: 2603, Cell Signalling Technology, Danvers, MA, USA). The day after, the membrane was reprobed for an hour at room temperature with an antibody to α-tubulin (cat. #: 2125, Cell Signalling Technology, Danvers, MA, USA), and AMPK and α-tubulin signals were revealed together. Signals were quantitated by densitometry using the ImageJ software (NIH, Bethesda, MD, USA).

### Blood glucose and insulin measurements

Two to three months old mice were fasted for 24 hours and blood was collected between 9am and 11am from the saphenous vein. For the refeeding experiments, mice were left at least one week to recover and were fasted again for 24 hours, refed between 9am and 11am and then, blood was collected 3 hours later. Blood glucose concentration was measured immediately when blood was collected using a One Touch II glucometer (LifeScan Canada Ltd., Burnaby, BC, Canada) and serum was frozen at −80°C. Serum insulin was measured using an ELISA kit (cat. #: EZRMI-13K, Linco Research Inc., MO, USA), according to the manufacturer's instructions.

### Thyroxine (T4) and corticosterone measurements

For T4 measurement, blood was collected from the saphenous vein in the afternoon and plasma was frozen at −80°C. Plasma T4 levels were measured using an EIA kit (cat. #: 07BC-1007, MP Biomedicals, Orangeburg, NY, USA) according to the manufacturer's instructions. For corticosterone measurements, 20 µl of blood was collected every 4 hours during a day, starting at 6am. In order to let the mice recover between harvests, blood was taken every 28 hours for 6 days. Serum from every sample was frozen at −80°C. Serum corticosterone levels were measured using an EIA kit (cat. #: 900-097, Assay Design, Ann Harbor, MI, USA) according to the manufacturer's instructions.

### Calorie restriction

Pre-weighed food pellets from Bioserv (Frenchtown, NJ, USA) were used for CR studies. Daily food intake of mice was determined for several weeks and averaged by genotype. Mice were adapted to the food for at least 1 month before CR started. SirT1 mutant mice and controls were assigned to AL or CR. Cohorts were matched for sex and litter when possible. Mice from CR groups were given 60% of average daily food intake of their corresponding genotype and mice from AL were given food unrestricted or 95% of average to prevent obesity. Age of mice was between 5 and 7 months when CR started and the diet was sustained for up to 44 weeks.
